# Superselective embolization for high-flow priapism refractory to medical and surgical treatments

**DOI:** 10.1186/s12894-020-00653-y

**Published:** 2020-06-30

**Authors:** Yonghua Bi, Mengfei Yi, Zepeng Yu, Xinwei Han, Jianzhuang Ren

**Affiliations:** Department of Interventional Radiology, The First Affiliated Hospital of Zhengzhou University, No.1, East Jian She Road, Zhengzhou, 450052 China

**Keywords:** Priapism, Embolization, Gelatin sponge particles, Polyvinyl alcohol particles

## Abstract

**Background:**

This study aimed to report long-term outcome of superselective embolization in patients with high-flow priapism refractory to medical and surgical treatments.

**Methods:**

From August 2011 until July 2016, 14 patients with high-flow priapism refractory to local treatments were treated and their charts were retrospective reviewed. Clinical evaluation, color Doppler ultrasonography, arteriography and selective embolization were performed. Follow up was performed in all patients. Fourteen men (18–63 years old) were enrolled, with priapism duration of 14 h to 28 days. Internal pudendal arteries or glutaea inferior arteriae were successfully embolized with gelatin sponge particles, polyvinyl alcohol particles or microcoils.

**Results:**

Pseudoaneurysm in right femoral artery was found in one case after intervention. The follow-up 1 week later showed that 13 patients were in good condition, the priapism diminished 1–7 days (mean 3.2 ± 0.5 days) after intervention, and 1 patient received second intervention. Mean follow-up was (range 10.8–69.6) months. One patient had recurrent priapism months after embolization and had his penis surgically removed for severe necrosis.

**Conclusions:**

Superselective embolization is safe and effective in high-flow priapism refractory local treatments, with a good long-term prognosis.

## Background

Priapism is characterized by a persistent penile erection (more than 4 h) continuing unrelated to sexual stimulation [1, 2], including the common low-flow priapism and high-flow priapism [1, 3, 4]. High-flow priapism is a rare condition, characterized by a prolonged and painless erection, which may permanently impair erectile function and should be treated timely to restore potency [5]. A fundamental aim of priapism management is try to relieve penile ischemia/anoxia and to prevent erectile dysfunction (ED) to achieve maximal preservation of sexual health [4].Surgery is the traditional method for high-flow priapism, however, this invasive treatment is less efficacious and associated with higher rates of ED compared with arterial embolotherapy [6]. Transarterial embolization is a useful alternative to surgery for high-flow priapism if local treatment fails [1, 7]. Early diagnosis of high-flow priapism and prompt intervention is essential to prevent future complications [8]. In this study, we report long-term outcome of superselective embolization in 14 cases with high-flow priapism refractory to medical and surgical treatments.

## Methods

### Patients

This retrospective study was approved by the Ethics Committee of The First Affiliated Hospital of Zhengzhou University. Informed consents were waived and all methods were carried out in accordance with the guidelines and regulations for clinical study. From August 2011 until July 2016, 14 patients with high-flow priapism refractory to medical or surgical treatment were reviewed in this retrospective chart review. Clinical evaluation, color Doppler ultrasonography, arteriography and selective embolization were performed. Follow up was performed in all patients.

### Procedure

After local anesthesia, Seldinger method was used to puncture femoral artery, a 5F vascular sheath was introduced. A 5F Cobra tube was introduced for the common iliac artery angiography, penile artery was catheterized by a micro catheter. Then embolization was performed with gelatin sponge particles, polyvinyl alcohol particles or microcoils. Second angiography was used to confirm the effect of emblolization.

## Results

Fourteen men were referred to our hospital with a complaint of a continuous erectile state, with a disease course of 14 h to 28 days and mean interval from onset to intervention of 18.8 ± 2.7 days. On admission to hospital, 2 showed normal general physical findings (Table 1). Implicated factors from chart review included, chronic myelogenous leukemia (*n* = 2), trauma in perineal region (*n* = 1), peudoaneurysm in right Internal pudendal artery (*n* = 1), urethral catheterization for urinary retention (*n* = 3), masturbation (*n* = 2), Chinese traditional medicine wine (*n* = 2), and German KINGBOX aphrodisiac (*n* = 1).

**Table 1:** The patients’ characteristics

Characteristics	Data
Patients, No.	14
Mean age, years	38.4 ± 4.2 (Range 18–63)
Mean interval from onset to intervention, Days	18.8 ± 2.7 (Range 1–40)
Recovery/days after intervention	3.2 ± 0.5 (Range 1–7)
Follow-up/years after intervention	3.1 ± 0.4 (Range 0.9–5.8)
Cause/ predisposition, No. (%)	
Urethral catheterization for urinary retention	3 (21.4%)
Chinese traditional medicine wine	2 (14.3%)
German KINGBOX aphrodisiac	1 (7.1%)
Trauma in perineal region	1 (7.1%)
Pseudoaneurysm	1 (7.1%)
None	4 (42.9%)
Previous failure treatments, No. (%)	
Aspiration therapy of corpus cavernosum	7 (50.0%)
External compression + local cold application	3 (21.4%)
Medication	2 (14.3%)
Medication + local cold application	2 (14.3%)

**Fig. 1:**
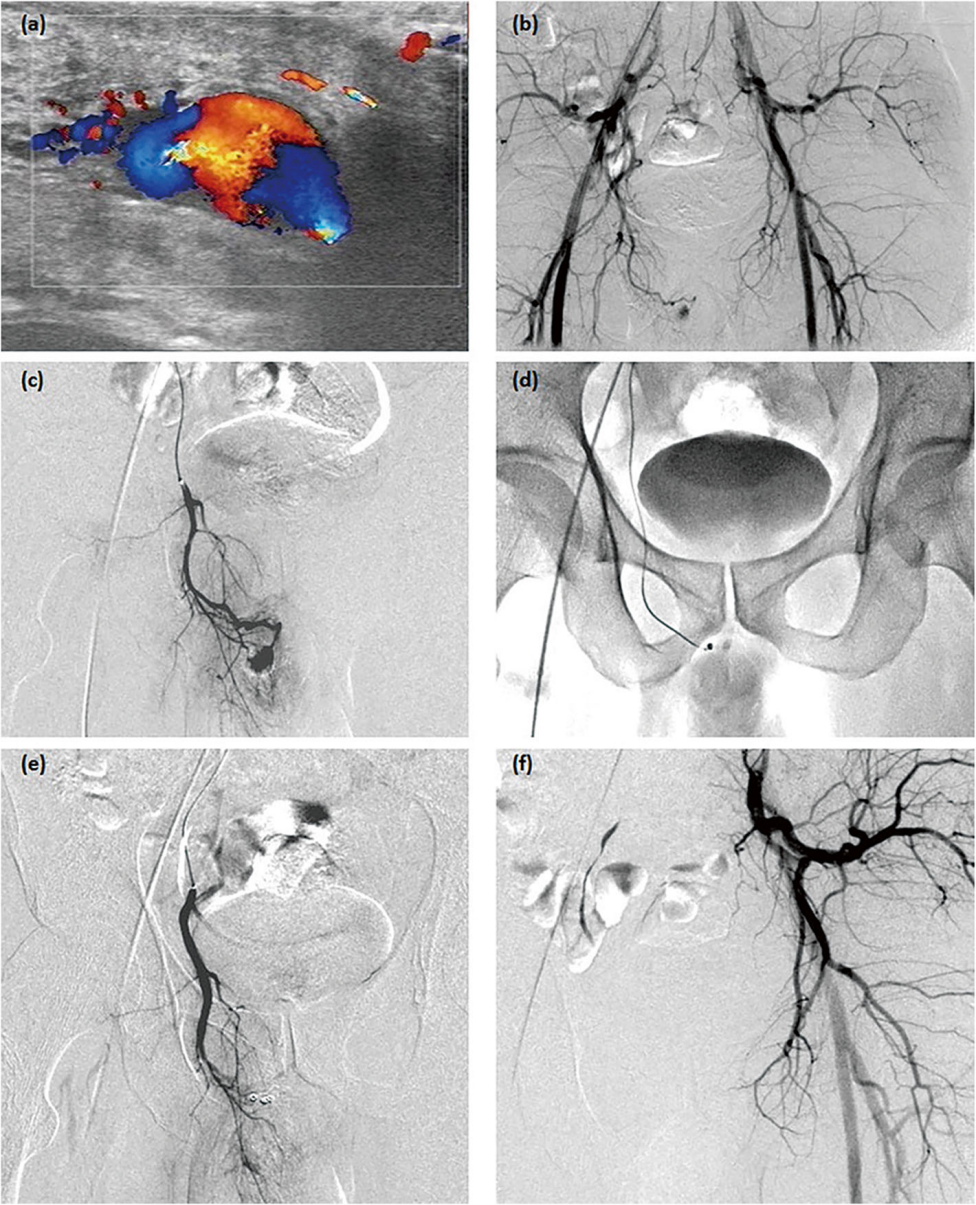
The treatment for case 1. One pseudoaneurysm was shown in right internal pudendal artery by ultrasonography (**a**) and angiography (**b**). Right internal pudendal artery was successfully catheterized (**c**), embolization of its branches was performed microcoils of 2 mm*2 cm (**d**), and pseudoaneurysm disappeared (**e**). The left internal pudendal artery was normal (**f**)

**Fig. 2:**
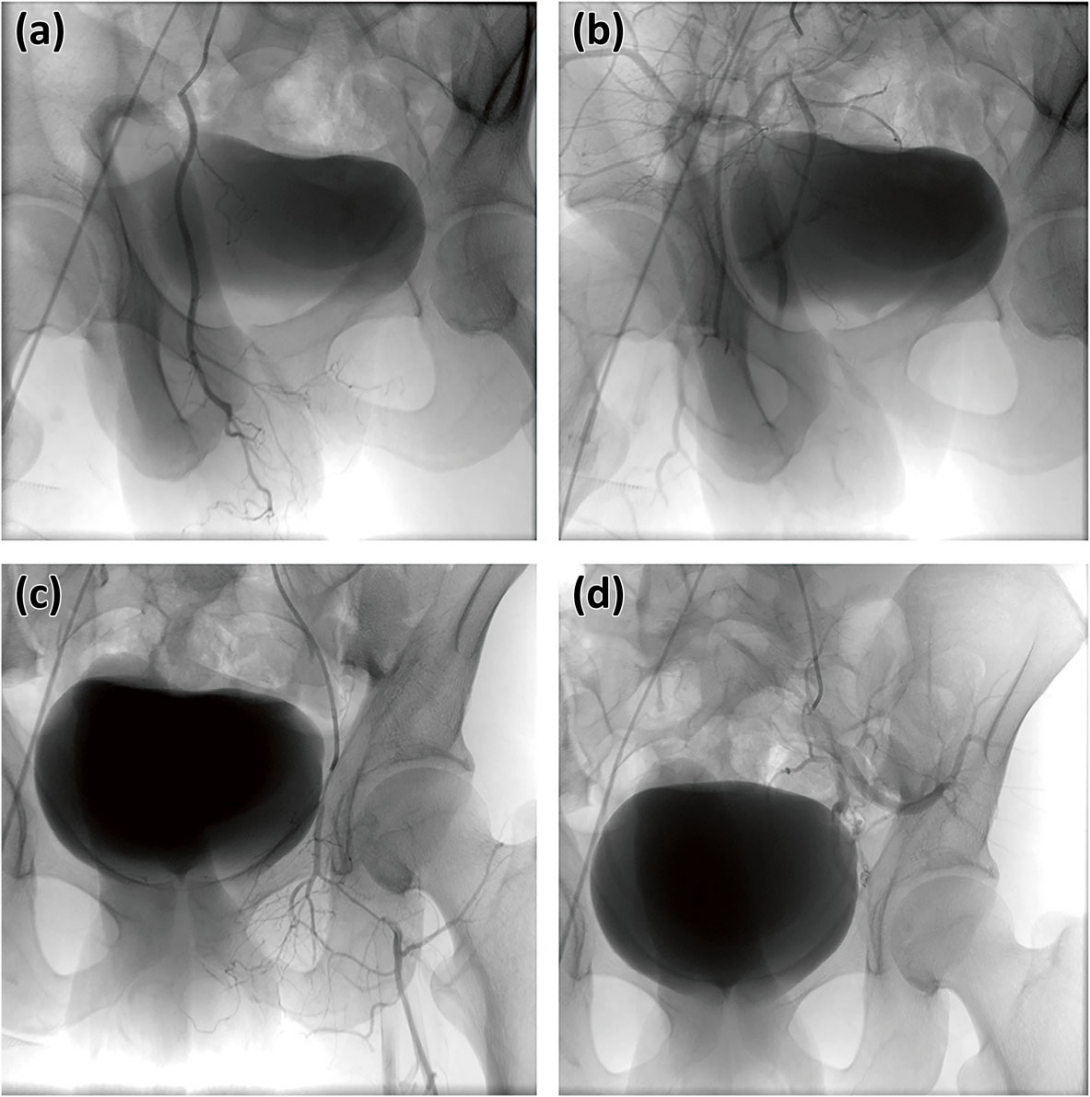
The treatment for case 2. Right internal pudendal artery was catheterized (**a**) and its branches were embolized with gelatin sponge particles of 350-560um (**b**). Left internal pudendal artery was catheterized and external iliac artery shown (**c**), microcoils of 3 mm*3 cm were used for embolization its branches (**d**)

**Fig. 3:**
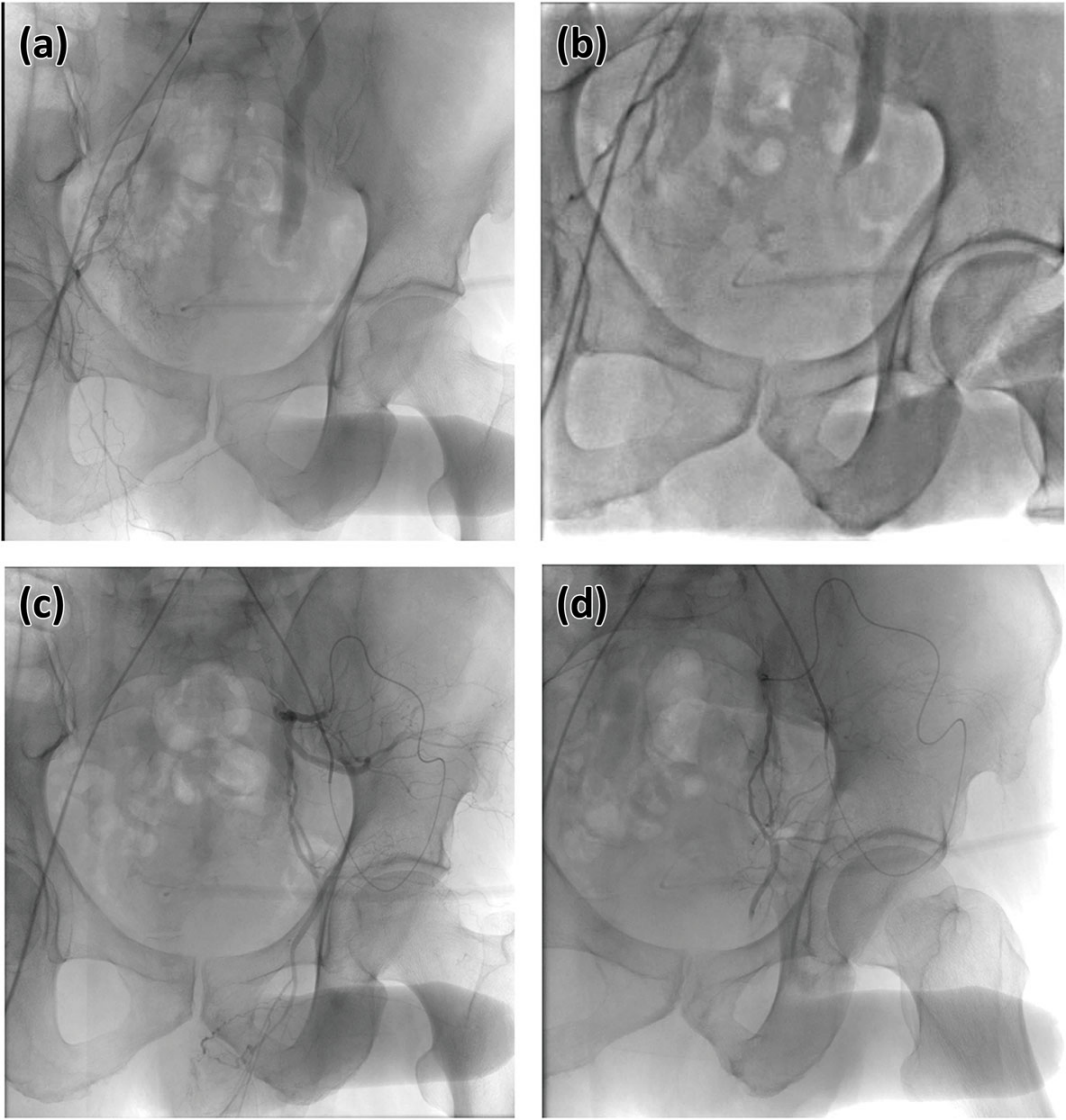
The treatment for case 3. Right internal pudendal artery was catheterized (**a**) and its branches were embolized with gelatin sponge particles of 350-560um (**b**). The left external iliac artery was catheterized for left internal pudendal artery (**c**), gelatin sponge particles of 350-560um were used for embolization its branches (**d**)

The patients were first examined by ultrasonography, which showed a high-velocity in cavernous arteries in all cases and one peudoaneurysm in the right internal pudendal artery (Fig. 1). A diagnostic angiographic study was performed before intervention. Internal pudendal arteries were successfully catheterized; embolization was performed with polyvinyl alcohol particles in 5 cases, gelatin sponge particles in 6 cases, and microcoils 3 cases (Figs. 1 and 2). The left external iliac artery was catheterized for arterial branches of the left internal pudendal artery in one case (Fig. 3). The patient with the pseudoaneurysm was embolized by two microcoils (2 mm*2 cm), and priapism was relieved on the second day after embolization.

No severe complications were seen immediately following embolization, but we did not perform detailed erectile function assessments Only the pseudoaneurysm in right femoral artery was found in one case after intervention. At the 1 week follow-up appointments, 13 patients reported intact erectile function, the priapism diminished 1–7 days (mean 3.2 ± 0.5 days) after intervention, and 1 patient received second intervention. Mean follow-up was ## (range 10.8–69.6) months. Recurrence was noted in one patient. Four years after the first treatment, the patient with priapism attributed to German KINGBOX aphrodisiac showed recurrence of priapism after took KINGBOX again. Color Doppler ultrasonography was used for diagnosis. Unfortunately, his penis had to be surgically removed because of severe necrosis due to lack of timely treatment.

## Discussion

Priapism is caused by a deregulation of penile detumescence, including low-flow (most common type), high-flow (very rare type) or stuttering priapism [9]. The high-flow type is usually caused by an arterial injury, developing a painless erection. Treatment options include mechanical, pharmacological, surgical method and intervention. Although surgery is the traditional treatment for high-flow priapism, it is more invasive and associated with higher incidence of ED compared to arterial embolotherapy [6]. Selective arterial embolization, first described in 1977 [10], is the current therapy of choice for high-flow priapism if local treatment fails, such as local cold application [11, 12].

Although reabsorbable materials have traditionally been the most commonly used, the choice of embolic agent remains inconsistent [6, 13] including autologous blood clot [14], gelatin sponge particles, polyvinyl alcohol [15], coils and N-butryl cyanide (NBCA) [6, 14], and Ethylene-vinyl alcohol copolymer (Onyx®) [3]. Autologous blood clot, a temporary occlusive agent, is very effective for treatment of high-flow priapism and permits restore normal blood flow after absorption [5, 6, 14, 16, 17]. Lloret et al. describe two cases of patients with high flow priapism treated by using non-reabsorbable materials of microcoil embolization [12]. In our study, gelatin sponge particles, polyvinyl alcohol [15], and microcoils were used for embolization. KINGBOX includes many substances featured in Chinese medicine, including Cordyceps sinensis, saffron crocus and more. There are also various compounds within Chinese medicinal wine that may be related to the phenomenon of priapism. We recognize the absence of firm evidence to link these drugs to the instances of priapism in our study, but our anecdotal clinical experience suggests that they may be related.

Complications of embolization may include ED, ischemia/necrosis, inflammation, and abscess formation [2, 9, 18]. It’s reported that the incidence of ED using nonabsorbable material is up to 39%, however, ED was only reported by the patient with the pseudoaneurysm of the right femoral artery.

There are limitations in our study. The sample is very few and the embolization materials are not uniform, so further study is still wanted. There are missing data on erectile function, and only two patients underwent blood gas analysis.

## Conclusions

Superselective transcatheter embolization is safe and effective in high-flow priapism refractory to medical or surgical treatments, with a good long-term prognosis.

## Data Availability

For further details, the corresponding author can be contacted.

## Abbreviation

ED: erectile dysfunction

## References

[1] Montague DK, Jarow J, Broderick GA (2003). American urological association guideline on the management of priapism. J Urol.

[2] Stock KW, Jacob AL, Kummer M, Zimmermann U, Steinbrich W (1996). High-flow priapism in a child: treatment with superselective embolization. AJR Am J Roentgenol.

[3] Chevallier O, Gehin S, Foahom-Kamwa A (2016). Ethylene-vinyl alcohol copolymer (onyx((R))) transarterial embolization for post-traumatic high-flow priapism. Quant Imaging Med Surg.

[4] Song PH, Moon KH (2013). Priapism: current updates in clinical management. Korean J Urol.

[5] Yesilkaya Y, Peynircioglu B, Gulek B, Topcuoglu M, Inci K (2013). Autologous blood-clot embolisation of cavernosal artery pseudoaneurysm causing delayed high-flow priapism. Pol J Radiol.

[6] Ozturk MH, Gumus M, Donmez H (2009). Materials in embolotherapy of high-flow priapism: results and long-term follow-up. Diagn Interv Radiol.

[7] O'Sullivan P, Browne R, McEniff N, Lee MJ (2006). Treatment of "high-flow" priapism with superselective transcatheter embolization: a useful alternative to surgery. Cardiovasc Intervent Radiol.

[8] Burns J, Rajendran S, Calder A, Roebuck D. High-flow priapism following perineal trauma in a child. BMJ Case Rep. 2015;2015. 10.1136/bcr-2014-208694 PMC4434374 25969486

[9] Castro RP, Hernandez PC, Casilda RR, Garcia JR, Tapia MJ (2010). Epidemiology of erectile dysfunction. Risk factors Arch Esp Urol.

[10] Wear JB, Crummy AB, Munson BO (1977). A new approach to the treatment of priapism. J Urol.

[11] Burnett AL, Bivalacqua TJ (2011). Priapism: new concepts in medical and surgical management. Urol Clin North Am.

[12] Lloret F, Martinez-Cuesta A, Dominguez P, Noguera JJ, Bilbao JI (2008). Arterial microcoil embolization in high flow priapism. Radiologia.

[13] Abujudeh H, Mirsky D (2005). Traumatic high-flow priapism: treatment with super-selective micro-coil embolization. Emerg Radiol.

[14] Numan F, Cantasdemir M, Ozbayrak M (2008). Posttraumatic nonischemic priapism treated with autologous blood clot embolization. J Sex Med.

[15] Sanchez-Lopez S, Gonzalez-Gomez S, Di Lizio-Miele K, Gonzalez-Gomez J (2017). High-flow priapism treated with superselective transcatheter embolization using polyvinyl alcohol particles. SAGE Open Med Case Rep.

[16] Akpinar S, Yilmaz G (2016). Autologous blood clot embolisation in posttraumatic high-flow priapism. Scott Med J.

[17] Cakan M, Altu Gcaron U, Aldemir M (2006). Is the combination of superselective transcatheter autologous clot embolization and duplex sonography-guided compression therapy useful treatment option for the patients with high-flow priapism?. Int J Impot Res.

[18] Belic D, Obrez-Oblak K (1990). Role of the tongue in the development of open bites. Zobozdrav Vestn.

